# Shaping Up for Battle: Morphological Control Mechanisms in Human Fungal Pathogens

**DOI:** 10.1371/journal.ppat.1003795

**Published:** 2013-12-26

**Authors:** David Kadosh

**Affiliations:** Department of Microbiology and Immunology, University of Texas Health Science Center at San Antonio, San Antonio, Texas, United States of America; Duke University Medical Center, United States of America

## Relationship between Fungal Morphology and Virulence

Many pathogenic and non-pathogenic fungi possess the ability to alter their morphology. The major fungal pathogens can grow vegetatively in multiple morphologies, including yeast, pseudohyphae, and hyphae. Yeast are typically round or oval-shaped single cells, while the filamentous morphologies, pseudohyphae and hyphae, are comprised of elongated cells attached end to end. Pseudohyphae are ellipsoidal in shape, possess constrictions at septal junctions, and are typically highly branched. Hyphae, in contrast, have parallel-sided walls, possess true septa (lacking constrictions), and often show a smaller filament width than pseudohyphae [1]. Pathogenic genera in the phylum zygomycota (e.g., *Absidia*, *Mucor*, *Rhizopus*, *Rhizomucor*, *Saksenaea*, and *Cunninghamella*) primarily grow as coenocytic (aseptate) hyphae or hyphae with only rudimentary septa. In addition, certain fungi (e.g., the pathogens *Aspergillus fumigatus* and *Penicillium marneffei*) also form distinctive reproductive structures on their hyphae, termed conidiophores, which generate asexual single-celled spores called conidia [2].

The ability to undergo a reversible transition from yeast to filamentous form is required for virulence of the major human fungal pathogen *Candida albicans*. Initial studies indicated that *C. albicans* mutants locked in either the yeast or filamentous form were avirulent in a mouse model of systemic candidiasis [3], [4]. More convincing evidence came from a later study using a strain in which the timing of the morphological transition could be controlled during the course of an infection [5]. When locked in the yeast form, this strain was avirulent, but the virulence defect could be reversed by allowing the strain to transition to filaments at various post-infection time points. An additional study provided further evidence by demonstrating that a strain showing increased hyphal formation could promote virulence in a mouse model of systemic candidiasis [6]. In *C. albicans* and other fungal pathogens, hyphal formation is known to be important for a variety of virulence-related processes, including tissue invasion, breaching of epithelial and endothelial cell layers, immune evasion, lysis of macrophages, biofilm formation, and thigmotropism (contact guidance) [7]–[9]. The *C. albicans* yeast form also plays an important role in several virulence-related processes, including adhesion to host cells, rapid dissemination through the bloodstream, and biofilm formation [10]. In addition, a recent genetic study has identified a variety of *C. albicans* mutants which are defective for infectivity but not morphogenesis (and vice versa) [11], suggesting that the relationship between filamentation and pathogenicity is not always precise and that yeast may be more important for pathogenesis than previously expected.

Interestingly, dimorphic fungal pathogens, such as *Histoplasma capsulatum*, *Coccidioides immitis*, *Paracoccidioides brasiliensis*, *P. marneffei*, *Blastomyces dermatitidis*, *Wangiella dermatitidis*, and *Sporothrix schenkii* are typically found as hyphae in the soil. Conidia derived from hyphal phase growth of these species are inhaled by the host (*S. schenkii* infections initiate after trauma) and differentiate to the infectious yeast form (or spherules for *C. immitis*) [2], [12]. Unlike *C. albicans*, these pathogens undergo a complete morphological conversion in vivo. Chemically blocking the hyphal–yeast transition renders *H. capsulatum* avirulent [13]. *H. capsulatum* yeast cells are known to multiply in macrophages, which promotes dissemination to several host organs, including the liver and spleen [14]. The large size of *B. dermatitidis* yeast cells has also been shown to prevent engulfment by polymorphonuclear neutrophils (PMNs) [15]. In addition, α-(1,3) glucan in the yeast cell wall of at least one of these species is known to block recognition by the macrophage β-glucan receptor dectin-1 [16].

Because of the importance of morphology for both virulence and virulence-related processes in a wide variety of pathogenic fungi, a significant amount of research has focused on determining the mechanisms by which morphological transitions are controlled. A comprehensive and detailed understanding of morphology regulation, in turn, may not only provide a better understanding of how certain fungal pathogens promote virulence in response to host environmental cues but also information leading to the development of novel and more effective antifungal strategies. Therefore, in the remainder of this review we will highlight and discuss several key morphological control mechanisms in pathogenic fungi. The recent discovery of these mechanisms has had a significant impact on the field, raising many new and interesting questions and opening up a variety of avenues for future research.

## Cell Type–Dependent and –Independent Morphological Regulatory Mechanisms

**Figure 1:**
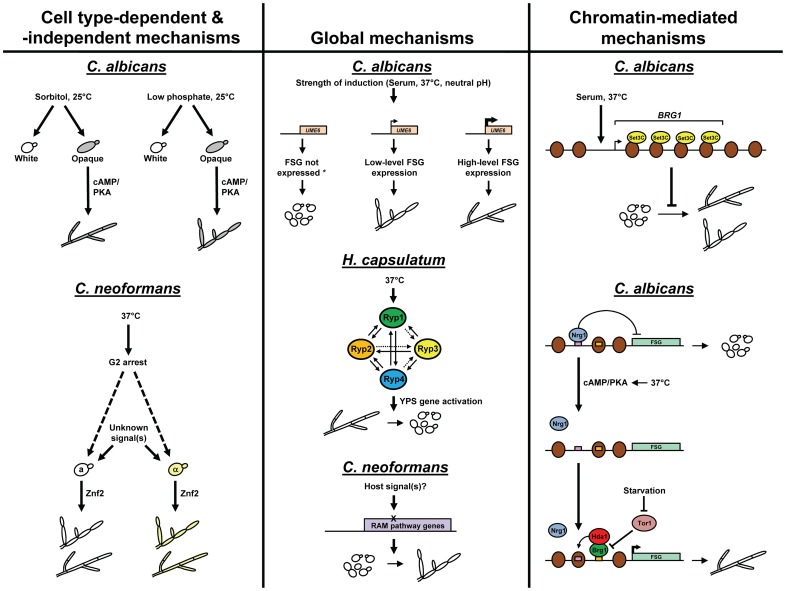
A comparison of molecular mechanisms used by human fungal pathogens to control morphology in response to environmental signals. Specific mechanisms are depicted for each species (adapted from and based on [6], [19], [20], [24]–[26], [28], [31], [33], [34], [36], [37], [40]). For *C. neoformans*, it is unclear at this point whether cell type–independent Znf2-mediated filamentation is induced by growth at 37°C and G2 arrest (dashed arrows). In the *H. capsulatum* mechanism, solid arrows between Ryp factors indicate ChIP-chip interactions and dashed arrows indicate indirect regulation. For the *C. albicans* Set3C mechanism, please note that *BRG1*, a strong activator of filamentation, is one of several key filamentous growth regulators whose transcription kinetics are modulated by the Set3C histone deacetylase complex during morphogenesis; in the presence of Set3C, filamentation is inhibited, but not completely abolished. In the *C. albicans* temporal chromatin alteration mechanism, please note that while Hda1 functionally associates with Brg1, a direct physical interaction has not yet been demonstrated; in addition, Tor1 inhibits Brg1 indirectly, by controlling *BRG1* transcriptional regulation. Please also note that not all morphological control mechanisms are depicted for every species. YPS = yeast-phase-specific, FSG = filament-specific genes, X = DNA mutation, brown circles = histones, *certain filament-specific genes may be expressed at a low basal level in the absence of induction.

Recent reports in both *C. albicans* and *Cryptococcus neoformans* have shed new light on the relationship among morphological transitions, mating, and cell type in pathogenic fungi. *C. albicans*, which normally grows as white cells, can undergo epigenetic switching to the mating-competent opaque cell type [17]. White cells (mating type **a**/α) are well known to generate filaments in response to a variety of environmental cues, including serum, 37°C, nutrient starvation, and neutral pH. Opaque cells, in contrast, do not filament in response to any of these conditions [18]. Interestingly, however, Si et al. have demonstrated that opaque cells (mating type **a**/**a**) can filament robustly at 25°C in the presence of distinct environmental conditions which do not induce white cells, including sorbitol and low-phosphate medium (Figure 1) [19]. Whole-genome transcriptional profiling revealed only limited overlap between gene expression programs during white versus opaque cell filamentation. In addition, while many of the same signaling pathways and transcriptional regulators control both white and opaque cell filamentation, opaque cells did not appear to respond to either the MAPK pathway or to the regulators Cph2 and Tec1. In a similar study, Guan et al. have identified Bcr1 as an opaque-specific transcriptional regulator of *C. albicans* filamentous growth [20]. How and why did an opaque-specific filamentation program, which responds to unique environmental cues, evolve in *C. albicans*? One possible explanation is that this program allows for opaque cell filamentation in specific host niches (e.g., the skin) which, in turn, could facilitate the interaction of mating partners. The natural environment in which this process occurs, as well as the role of opaque-specific filamentation in pathogenesis and tissue invasion, remains to be elucidated.

In contrast to *C. albicans*, *C. neoformans* typically grows in the yeast form but can undergo a transition to filamentous cells during bisexual (**a**-α) and unisexual (mostly α–α) mating, as well as monokaryotic fruiting (either **a** or α) [21]–[24]. A recent study indicates that expression of the key transcriptional regulator Znf2, can drive *C. neoformans* filamentation irrespective of mating type and serotype (Figure 1) [25]. A Znf2-expressing strain generated filaments during infection in vivo and was avirulent in a mouse model, confirming the importance of the yeast form for *C. neoformans* pathogenicity. Although Znf2 is known to be induced by V8 medium in vitro, the specific environmental signals responsible for cell type–independent Znf2 induction and the *C. neoformans* morphological transition are unknown. However, one possible clue comes from the recent identification of a mating-independent mechanism for *C. neoformans* monokaryotic hyphal production [24]. This pathway is induced by high temperature (37°C) and specifically associated with G2 cell cycle arrest. Because pigeon droppings (which can reach high temperatures in compost piles) are known to represent environmental reservoirs for *C. neoformans*, one possible theory is that exposure to the body temperature of warm-blooded hosts primes *C. neoformans* cells for G2 arrest. However, at the current time, very little is known about the precise role of mating- and cell type–independent filamentation mechanisms in this pathogen.

## Global Mechanisms that Control Morphology Determination

Several recent advances have been made that significantly improve our understanding of how fungal pathogen morphology is determined at the global level. In *C. albicans*, expression levels of a key filament-specific transcriptional regulator gene, *UME6*, were shown to be necessary and sufficient to determine morphology [6]. In the absence of *UME6* expression, cells grew as yeast. Low levels of *UME6* specified cells mostly in the pseudohyphal morphology, and at high *UME6* expression levels, cells grew as a nearly complete hyphal population (Figure 1). Whole-genome transcriptional profiling indicated that both the level and duration of *UME6* expression play important roles in determining *C. albicans* morphology by controlling the expression of overlapping sets of filament-associated target genes [26]. Interestingly, genes associated with growth in the pseudohyphal morphology appeared to represent a subset of those associated with hyphae and were generally expressed at lower levels. These results suggested that all three *C. albicans* morphologies are determined by a common dosage-dependent mechanism and raised the intriguing possibility that pseudohyphae may represent an intermediate morphology between yeast and hyphae. However, this hypothesis is still somewhat controversial since the principles for growth in each morphology are very different [27].

In *H. capsulatum*, a global transcriptional network has very recently been shown to control the hyphal–yeast transition in response to temperature (Figure 1) [28]. This network is comprised of four transcriptional regulators (Ryp1-4), all of which are required for yeast phase growth at 37°C. In addition to controlling the expression of one another by a positive feedback circuit (Ryp1-3 also form a complex), both whole-genome ChIP-Chip and expression profiling experiments indicate that the Ryp factors associate with and function to regulate the expression of a large common set of genes, including many virulence factors and the majority of yeast-phase specific genes. The identification of this network raises many new questions and opens up a variety of avenues for future research. What signaling mechanisms control activation of the network in response to temperature? How exactly do specific target genes in the network promote *H. capsulatum* virulence and/or macrophage survival in the host? Do other fungal pathogens possess similar networks to control morphology (and associated virulence attributes) in response to environmental cues such as those encountered in the host? A hypha-specific transcriptional regulator that is both necessary and sufficient to drive hyphal growth as well as inhibit conidiation and yeast growth has recently been identified in *P. marneffei* [29]. The *DRK1* hybrid histidine kinase is also known to function as a global regulator of morphology and virulence in both *B. dermatitidis* and *H. capsulatum* [30]. These findings suggest that additional dimorphic fungal pathogens also possess global mechanisms important for morphology determination.

In *C. neoformans*, a mechanism that responds to DNA mutation frequency has recently been shown to control the transition from yeast to pseudohyphae upon passage through the amoeba (Figure 1) [31]. This mechanism is specifically triggered by DNA mutations in genes of the RAM signaling pathway. Interestingly, the resulting pseudohyphae were temperature-sensitive and significantly attenuated for virulence in a mouse model, which is counter to the hypothesis that passage of *C. neoformans* through the amoeba selects for more virulent strains in the mammalian host [32]. Importantly, these findings also suggest a direct link between a host evasion mechanism, increased DNA mutation frequency, and morphology determination in a fungal pathogen. The evolutionary advantage of *C. neoformans* pseudohyphal conversion in the amoeba is unclear at this point, although it may provide a means to escape phagocytosis. How and why this mechanism appears to be more active in the amoeba versus the mammalian host also remains to be determined.

## Chromatin-Mediated Morphological Control Mechanisms

Several new discoveries in *C. albicans* suggest that chromatin-mediated mechanisms also play an important role in controlling morphological transitions of pathogenic fungi. The Set3/Hos2 histone deacetylase complex (Set3C) has been shown to associate with highly transcribed genes and functions as a repressor of the *C. albicans* yeast-filament transition (Figure 1) [33], [34]. This complex also specifically modulates the transcription kinetics as well as transient expression of several key regulators of filamentous growth (*NRG1*, *EFG1*, *BRG1*, and *TEC1*). Additional chromatin-modifying activities known to play an important role in *C. albicans* morphogenesis include the Rtt109 histone acetyltransferase, the Set1 histone methyltransferase, and components of the Swi/Snf chromatin remodeling complex [35]. Another study has demonstrated that *C. albicans* hyphal development and the expression of hypha-specific genes requires two temporally linked alterations in promoter chromatin (Figure 1) [36]. In the initiation phase, Nrg1, an important transcriptional repressor of filamentous growth, is released from hypha-specific promoters as a result of activation of the cAMP-PKA pathway. In order to maintain hypha-specific gene expression and develop extended hyphae, Hda1 histone deacetylase is subsequently recruited to the promoter by the transcriptional regulator Brg1 in the presence of reduced Tor signaling [37]. This maintenance phase only occurs in the absence of Nrg1 and, when completed, blocks future access of Nrg1 to the promoter; Nrg1 binding sites were shown to be occluded by nucleosome repositioning, leading to a “hyphal chromatin state.” Interestingly, Brg1 also targets Ume6, which appears to function in a positive feedback loop to maintain hypha-specific gene expression and hyphal growth [37]. The complete set of factors which modulate *C. albicans* chromatin-mediated morphology regulation in response to specific host environmental cues has yet to be determined. In addition, the extent to which chromatin-mediated mechanisms control morphology in other fungal pathogens is unclear at this point. However, this possibility is suggested by a recent demonstration that Gcn5 histone acetyltransferase controls a variety of virulence-related processes in *C. neoformans* [38].

## Perspectives and Future Directions

As indicated above, human fungal pathogens have evolved a wide range of molecular mechanisms to control morphological transitions in response to diverse environmental signals. For fungal pathogens that normally reside in mammals or are transmitted by human-to-human contact or via fomites (e.g., *Candida* spp., dermatophytes, and *Pneumocystis jirovecii*), these mechanisms most likely evolved via direct selection pressure from the host. For human fungal pathogens with major reservoirs in the environment, morphological control mechanisms may have evolved via selection in heterologous hosts or as components of virulence cycles which are not yet fully understood; in addition, certain traits which are selected for by evolution in specific environmental niches may also confer a selective advantage in the host environment (e.g., compost piles, which are reservoirs for *A. fumigatus*, are naturally heated to high temperatures ≥37°C and, as a consequence, this species may have evolved traits that confer a selective advantage in both the compost pile environment as well as the mammalian host). In either case, the mechanisms described in this article, most of which have been discovered only in the past few years, may represent just the tip of the iceberg. Although an anti-sense–mediated mRNA stability mechanism has recently been shown to control a key regulator of *C. albicans* filamentous growth [39], in general, very little is known about post-transcriptional or translational mechanisms that may play a role in directing morphological transitions and/or virulence in pathogenic fungi. Knowledge of post-translational mechanisms that control the stability, localization, and/or modification of key morphology regulators is also particularly lacking. In addition, because detailed morphological control mechanisms have been described for only a few fungal pathogens, the extent to which they are evolutionarily conserved remains unknown. Ultimately, future studies which address these and other important questions are likely to significantly improve our understanding of how fungal pathogens control morphology and virulence in response to host environmental cues and may provide information leading to the development of new and more effective antifungal strategies.
